# Prevalence and healthcare utilization in managing herpes zoster in primary care: a retrospective study in an Asian urban population

**DOI:** 10.3389/fpubh.2023.1213736

**Published:** 2023-09-15

**Authors:** Xin-Bei Valerie Chan, Ngiap Chuan Tan, Mark Chung Wai Ng, Ding Xuan Ng, Yi Ling Eileen Koh, Wai Keong Aau, Chirk Jenn Ng

**Affiliations:** ^1^SingHealth Polyclinics, Singapore, Singapore; ^2^SingHealth Duke-NUS Family Medicine Academic Clinical Programme, Singapore, Singapore

**Keywords:** herpes zoster, prevalence, healthcare, primary care, prevention, treatment

## Abstract

Herpes zoster (HZ) causes significant morbidity, particularly in older adults. With the advent of a recombinant zoster vaccine, HZ is potentially preventable. However, data on HZ burden and healthcare utilization in primary care populations remains scarce. This study described the prevalence and healthcare utilization in managing HZ in a developed community. A retrospective database review was conducted across a cluster of 8 public primary care clinics in urban Singapore. Data of multi-ethnic Asian patients with a diagnosis code of “herpes zoster” from 2018 to 2020 was extracted from their electronic medical records. Socio-demographic, clinical, visitation, medical leave, prescription, and referral data were analyzed. A total of 2,987 out of 737,868 individuals were diagnosed with HZ over 3 years. The mean age was 59.9 (SD + 15.5) years; 49.2% were male; 78.5% Chinese, 12.2% Malay, and 4.1% Indian. The prevalence was 221, 224, 203 per 100,000 persons in 2018, 2019, and 2020, respectively. The 70 to 79-year age group had the highest prevalence (829/100,000) across 3 years. Oral acyclovir (median daily dose 4,000 mg; median duration 7 days) and topical acyclovir were prescribed in 71.6 and 47.6%, respectively. Analgesia prescribed were gabapentin (41.0%), paracetamol combinations (30.1%), oral NSAIDs (23.7%), opioids (6.0%), and tricyclic antidepressants (1.9%). Most individuals consulted only once (84.3%); 32.7% of them required medical leave and 5.6% had more than 7 days of absenteeism. HZ-related referrals to the hospital were required in 8.9% (4.9% emergency, 2.8% ophthalmology). The findings of this study suggest a need for HZ vaccination among older age groups. Visitation and referral rates were low. The use of topical acyclovir was uncovered, and further research should evaluate the underlying reasons, benefits, and harms of such practice. The use of analgesia combinations may be explored further.

## Introduction

HZ is a common infectious disease encountered in primary care, especially among older adults. The estimated lifetime risk of HZ in the general population is 30% (1, 2), and it increases sharply after 50 years of age, reaching a lifetime risk of 50% at age 85 years (3, 4). With aging populations, the average HZ incidence of 3–10/1,000 person-years in Asia Pacific countries is rising at around 5% per year (5).

HZ causes significant morbidity. Nearly 1 in 4 cases (23%) develop HZ-related complications (6). A systematic review established that the risk of postherpetic neuralgia ranges from 5% to more than 30%, while the risk of herpes zoster ophthalmicus ranges from 10% to 14.9% (7). HZ-related pain is known to adversely affect quality of life, function, and productivity (8, 9). HZ patients may also develop otologic (Ramsay Hunt syndrome), neurological (meningoencephalitis, motor neuropathy, transverse myelitis, cerebral vasculitis, and cranial palsies), and dermatological complications (bacterial superinfection) (3, 10).

HZ-related morbidity may result in significant healthcare utilization. Healthcare utilization refers to the use of a healthcare service, procedure, device, or drug for the purpose of maintaining one’s health and well-being, preventing and/or treating health problems, or obtaining information about one’s health status and prognosis (11). In a South Korean study, the most utilized healthcare resource was visits to a primary care physician (98.7% of patients), followed by visits to a specialist (55.0%), hospitalizations (32.5%), and visits to the emergency department (17.9%) (8). In other studies, HZ-associated hospitalization rates ranged from 3.4% to 4.79% (12, 13). Patients with HZ required an average of 3.4–5.7 outpatient consultations (13–15) and most were prescribed antivirals and analgesia (13, 15, 16). HZ-related absenteeism ranged from 2.67 ± 3.89 days to 11.3 ± 5.7 days in different countries (17, 18).

HZ may be prevented with HZ vaccination. At present, both the live attenuated zoster vaccine and recombinant zoster vaccine (RZV) are available by patient request at travel clinics within public hospitals and a few private primary care clinics. However, public primary care clinics in Singapore do not provide HZ vaccination. Various countries have adopted RZV into their national guidelines for HZ prevention (19), but HZ vaccination has yet to be incorporated into the National Adult Immunization Schedule in Singapore (20). Prior Singaporean studies conducted in tertiary settings reported increased age as a risk factor for HZ and post-herpetic neuralgia (16, 21, 22). An economic analysis concluded that HZ vaccination is likely to be cost-effective for adults over 50 years of age in Singapore (23). However, the simulation model used incidence rates from other countries due to the scarcity of local data, and economic costs reported in a dermatological center. A knowledge gap in the disease burden and healthcare utilization of HZ remains among local primary care physicians.

By evaluating the burden of HZ and its utilization of healthcare resources, primary care providers can make informed decisions on the necessity of targeted HZ vaccination and identify any gaps in current management practices. Therefore, this study aimed to describe the prevalence and healthcare utilization of HZ in a Singapore primary care setting. The secondary objective was to identify factors that may be associated with HZ. The findings of this study may inform decisions related to primary prevention and management of HZ.

## Materials and methods

### Study design

A cross-sectional population-based study was conducted via a retrospective database review, which provided a large sample size to assess disease burden. The adopted study design allowed for the estimation of actual healthcare utilization since patients were managed according to routine clinical practice in a real-world setting. In addition, the use of a database provided the large amount of data required to identify patterns and potential associations between HZ and various factors.

### Data source

Data was sourced from the SingHealth-IHiS Electronic Health Intelligence System (eHints), a repository that integrates information from administrative, clinical, and ancillary healthcare transactional systems. eHints was established in 2009 and contains data from the SingHealth cluster, one of three public healthcare clusters in Singapore, that covers the Eastern part of the country. All Singapore residents have the option to visit any public primary care clinic regardless of their residential address. These clinics offer subsidized care to citizens and permanent residents, with higher subsidies for children and the older adults. Data was collected from all eight public primary care clinics within the SingHealth cluster.

Data on demographic characteristics, clinical diagnoses, prescriptions, clinic attendances, and referrals were extracted. Clinical diagnoses were coded using the ICD-10 classification. Clinic attendances occurred exclusively in the form of in-person visits. Telephone and home visits were not available at public primary care clinics during the study period. All referrals were made electronically. From the “Clinical Document (Referrals Only)” section of the eHints database, the following fields were extracted: visit number, referral destination, and referral indication. Medical certificates were issued electronically. Data regarding the visit number and duration of medical leave given each visit was extracted from the “Medical Certificate” section of the eHints database.

A trusted third party was appointed by the healthcare cluster to perform data de-identification. The de-identified data was passed to the study team via secure file transfer protocol. As secondary data without personal identifiers was used, informed consent was not required, and the study was exempted from approval by the SingHealth Centralised Institutional Review Board (Reference number: 2021/2709).

### Data quality checks

The de-identified data was checked for missing and out-of-range values, duplication as well as mislabeling prior to merging. Six individuals were excluded for missing values and another two individuals were excluded for outlying values. After the data was cleaned and merged, further checks on data consistency were conducted.

### Selection of cases

The study population comprised of all individuals, aged 21 years and above, who attended a cluster of eight public primary care clinics in eastern Singapore from 1 January 2018 to 31 December 2020. Cases were defined as individuals who had one or more visits with a diagnosis code of HZ between 1 January 2018 to 31 December 2020.

### Associated factors

The factors of interest were age, gender, ethnicity, and chronic medical conditions that are prevalent in the primary care population. These factors were studied to identify high-risk groups to facilitate the development of targeted prevention and treatment strategies. Increased age, female gender, and chronic medical conditions have been reported to increase the risk of HZ (24). Compared to Caucasian individuals, African-American individuals were shown to have a lower risk of contracting HZ, while Asian Americans had a similar risk (24). The three major ethnic communities in Singapore were examined—Chinese, Malay, and Indian.

Chronic conditions identified from the literature as potential risk factors included asthma (24–26), chronic kidney disease (27, 28), chronic obstruction pulmonary disease (29, 30), depression (31, 32), diabetes mellitus (33, 34), hyperlipidemia (32), hypertension (35), hypothyroidism (36), ischemia heart disease (37), osteoarthritis (32), and rheumatoid arthritis (38). Including the former conditions, we selected 21 diseases covered under the Chronic Disease Management Programme (CDMP), a healthcare financing scheme that allows the use of compulsory medical savings (Medisave) for outpatient treatment of common chronic conditions. The 21 chronic diseases were anxiety, asthma, benign prostatic hyperplasia (BPH), bipolar disorder, chronic kidney disease (CKD), chronic obstructive pulmonary disease (COPD), dementia, depression, diabetes mellitus, epilepsy, hyperlipidemia, hypertension, ischemic heart disease (IHD), osteoarthritis, osteoporosis, Parkinson’s disease, prediabetes, psoriasis, rheumatoid arthritis, schizophrenia, and stroke. Hypothyroidism, although not part of CDMP, was also selected since it was identified as a potential risk factor from the literature (36). When examining the association between each chronic condition and HZ, cases were included only if there were any visit(s) for each chronic condition that occurred before the earliest recorded HZ visit. A look-back period from 1 January 2015 to 31 December 2020 was used to check for visits with diagnosis codes of the studied chronic conditions. The diagnosis codes used to ascertain HZ and the studied chronic conditions are specified in the Supplementary material.

### Statistical analysis

The yearly prevalence of HZ for years 2018, 2019, and 2020 was calculated as the number of cases that occurred per 100,000 persons who visited the eight public primary care clinics. The calculation was based on the formula for period prevalence, defined as the proportion of people in a population having disease over a period of time (39). Individuals who had visits with a diagnosis code of HZ in 2018, 2019, and/or 2020 would be counted as cases for the respective years.

Given HZ occurs more commonly in older adults (5), the prevalence by age was examined to determine the burden of HZ in older age groups. To determine the number of unique cases across each age group, each case was identified by the index HZ visit, which was the earliest recorded visit with a diagnosis code for HZ. The date of the index visit was considered the date of diagnosis of HZ.

For healthcare utilization, the number of clinic attendances per episode of HZ, referrals made to tertiary care for further management of HZ, medications prescribed for HZ, and days of medical leave taken per episode of HZ were evaluated using frequency distributions. The denominator used for all the above was the total number of cases, except for days of medical leave where the denominator was the number of cases requiring medical leave. For each case, all visits with a diagnostic code of HZ were assumed to belong to a single HZ episode.

In determining associated factors of HZ, the independent variables used were age, gender, ethnicity, and the 22 chronic diseases. The dependent variable was HZ occurrence. Age was analyzed as a dichotomous variable (aged below 50 years, aged 50 years and above). The cut-off of 50 years old was chosen as it is known that HZ risk increases sharply onwards from this age (3). Pearson’s chi-square test of independence was used to test for the presence of associations between the independent variables and HZ. For factors that demonstrated an association with HZ, logistic regression was performed to determine the strength of the association.

The frequencies of each chronic condition were determined among the cases, non-cases, and total population. Chronic conditions with fewer than 10 HZ cases were excluded from the analysis—Parkinson’s disease, epilepsy, bipolar disorder, schizophrenia, rheumatoid arthritis, and psoriasis. Univariate analysis was conducted between each of the remaining conditions and HZ. Factors that showed a statistically significant association with HZ (*p* < 0.05) were added to a multivariate model. Chronic conditions shown to be associated with HZ occurrence in literature were added to the multivariate model regardless of whether they demonstrated a significant relationship with HZ in univariate analysis. Multivariate logistic regression was used to calculate adjusted odds ratios (aOR) with 95% confidence intervals (95% CI). Checks for multicollinearity were undertaken by assessing variance inflation factors (VIF) for independent variables and performing correlation matrix analysis. VIF for all independent variables was <2.2, and pairwise correlation coefficients were < 0.7, suggesting that multicollinearity was not a concern, using thresholds of 5 and 0.8, respectively (40).

Statistical analysis was performed using SPSS version 28.0 (IBM SPSS Statistics for Windows, Version 28.0. Armonk, NY: IBM Corp.).

## Results

**Figure 1:**
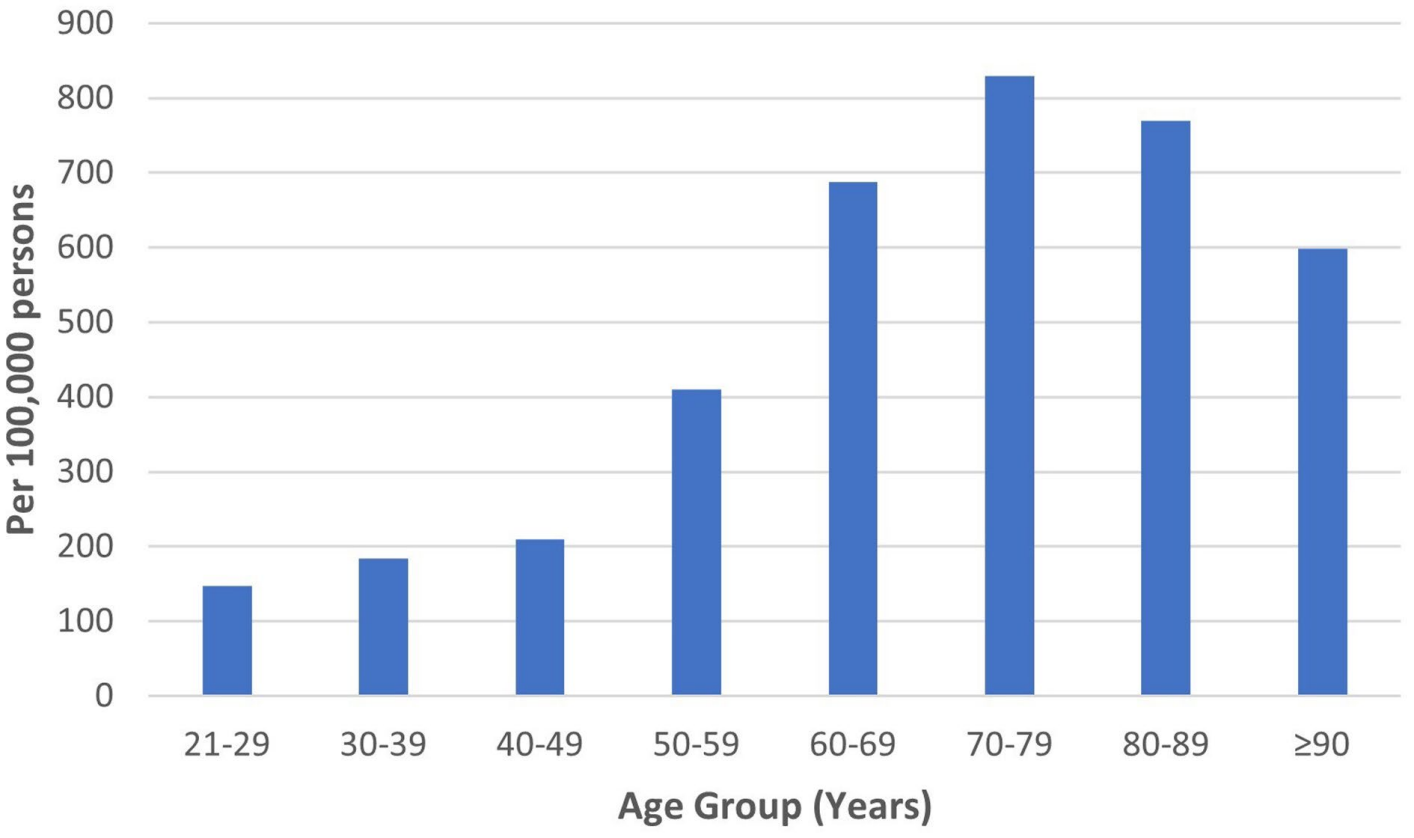
Prevalence of herpes zoster by age in the study population from 2018 to 2020.

HZ was diagnosed among 2,987 of 737,868 individuals who visited the study sites over 3 years. The mean age of HZ diagnosis was 59.9 ± 15.5 years. The median age of HZ diagnosis was 62.0 years. Males constituted 49.2%. The ethnicity distribution was as follows: 78.5% Chinese, 12.2% Malay, and 4.1% Indian. HZ prevalence was 221, 224, and 203 per 100,000 persons in 2018, 2019, and 2020, respectively, with a mean yearly prevalence of 216 per 100,000 persons across 3 years (Table 1). The highest HZ prevalence of 829 per 100,000 persons occurred in the 70 to 79-year age group (Figure 1).

**Table 1:** Prevalence of herpes zoster in the study population.

*Yearly prevalence of herpes zoster*			
Year	Cases	Population	Prevalence per 100,000 population
2018	1,017	459,687	221
2019	1,079	480,955	224
2020	927	457,443	203

*Prevalence of herpes zoster by age*			
Age at index visit (years)	Cases	Population	Prevalence per 100,000 population
21–29	178	120,709	147
30–39	227	123,045	184
40–49	243	115,499	210
50–59	565	137,900	410
60–69	944	137,266	688
70–79	590	71,207	829
80–89	211	27,392	770
≥ 90	29	4,850	598
Total	2,987	737,868	405

The majority of HZ cases (91.1%) were managed entirely in the primary care setting. Most cases consulted only once for HZ (84.3%) (Table 2). Referrals were required for 8.9% of cases, most commonly to emergency (4.9%) and ophthalmology (2.8%) departments. Of 146 referrals to emergency, 73.3% were for suspected herpes zoster ophthalmicus. The remainder of emergency referrals were for suspected Ramsay Hunt syndrome, disseminated zoster, HZ occurring in the immunosuppressed, zoster encephalitis, pain control, and secondary bacterial infections. Of the 13 referrals in the “Others” category, two were to obstetrics and gynecology for zoster in pregnancy, and the rest to a myriad of specialties based on the location of zoster rash.

**Table 2:** Healthcare utilization of HZ cases.

	Cases (*n* = 2,987)	
	Number	Percentage
***Clinic visits per episode of herpes zoster***		
1	2,518	84.3
≥2	469	15.7
Range	1–8	
Median (IQR)	1 (1–1)	
***Referrals made for further management of herpes zoster***		
Emergency	146	4.9
Ophthalmology	83	2.8
Otolaryngology	16	0.5
Dermatology	15	0.5
Infectious disease	3	0.1
Neurology	3	0.1
Others	13	0.4
***Medications prescribed for herpes zoster***		
**Antiviral**		
Oral acyclovir	2,138	71.6
Topical acyclovir	1,422	47.6
**Analgesia**		
Gabapentin	1,224	41.0
Paracetamol combinations	898	30.1
NSAIDs (oral)	708	23.7
Opioids	178	6.0
Lidocaine (topical)	86	2.9
TCAs	58	1.9
NSAIDs (topical)	40	1.3
***Days of medical leave taken per episode of herpes zoster*** ^**a**^		
≤7	811	83.0
>7	166	17.0
Range	1–23	
Median (IQR)	5 (2–7)	

IQR, Interquartile Range; NSAIDs, Non-Steroidal Anti-Inflammatory Drugs; TCAs, Tricyclic Antidepressants.

a Denominator is 977 cases requiring medical leave.

For treatment of HZ, oral and topical acyclovir were prescribed in 71.6% and 47.6%, respectively (Table 2). The median daily dose for oral acyclovir was 4,000 mg, given as 800 mg 5 times daily, and the median duration was 7 days. The commonly prescribed analgesics were gabapentin (41.0%), paracetamol combinations (30.1%), and oral non-steroidal anti-inflammatory drugs (NSAIDs) (23.7%).

Medical leave was required in 977 cases (32.7%). In terms of absenteeism, 17.0% of those requiring medical leave (5.6% of all cases) incurred more than 7 days.

In multivariate analysis, age, ethnicity, and asthma demonstrated the strongest relationships with HZ (Table 3). A person aged 50 years and above would have more than three times the odds of being diagnosed with HZ (aOR 3.16, 95% CI 2.87–3.48, *p* < 0.001). Chinese and Malays were more likely to develop HZ than Indians (Chinese: aOR 1.80, 95% CI 1.50–2.16, *p* < 0.001; Malay: aOR 1.75, 95% CI 1.42–2.14, *p* < 0.001). The odds of HZ were increased by more than 30% among patients with asthma (aOR 1.35, 95% CI 1.12–1.62, *p* = 0.002). Individuals with hypertension and osteoarthritis were also more likely to be diagnosed with HZ (hypertension: aOR 1.24, 95% CI 1.11–1.38, *p* < 0.001; osteoarthritis: aOR 1.25, 95% CI 1.12–1.38, *p* < 0.001). CKD and diabetes were negatively associated with HZ (CKD: aOR 0.71, 95% CI 0.62–0.80, *p* < 0.001; diabetes: aOR 0.88, 95% CI 0.79–0.98, *p* = 0.024).

**Table 3:** Factors associated with HZ in the study population.

	No. (%)			Unadjusted OR (95%CI)	*p*-value	Adjusted OR (95%CI)	*p*-value
	Total (*n* = 737,868)	Non-cases (*n* = 734,881)	Cases (*n* = 2,987)				
**Age (years), median (IQR)**	50 (35–63)	50 (35–63)	62 (52–70)	–	–	–	–
**Age (years)**							
< 50	359,220 (48.7)	358,572 (99.8)	648 (0.2)	1	–	1	–
≥ 50	378,648 (51.3)	376,309 (99.4)	2,339 (0.6)	3.44 (3.15–3.75)	<0.001	3.16 (2.87–3.48)	**<0.001**
**Gender**							
Female	402,111 (54.5)	400,594 (99.6)	1,517 (0.4)	1	–	1	–
Male	335,757 (45.5)	334,287 (99.6)	1,470 (0.4)	1.16 (1.08–1.25)	<0.001	1.19 (1.11–1.28)	**<0.001**
**Ethnicity**							
Indian	59,043 (8.0)	58,920 (99.8)	123 (0.2)	1	–	1	–
Chinese	517,264 (70.1)	514,920 (99.5)	2,344 (0.5)	2.18 (1.82–2.62)	<0.001	1.80 (1.50–2.16)	**<0.001**
Malay	98,903 (13.4)	98,538 (99.6)	365 (0.4)	1.77 (1.45–2.18)	<0.001	1.74 (1.42–2.14)	**<0.001**
Others	62,658 (8.5)	62,503 (99.8)	155 (0.2)	1.19 (0.94–1.51)	0.154	1.44 (1.14–1.83)	**0.003**
**Anxiety**							
No	731,551 (99.1)	728,579 (99.6)	2,972 (0.4)	1	–	1	–
Yes	6,317 (0.9)	6,302 (99.8)	15 (0.2)	0.58 (0.35–0.97)	0.038	0.62 (0.37–1.03)	0.063
**Asthma**							
No	715,405 (97.0)	712,538 (99.6)	2,867 (0.4)	1	–	1	–
Yes	22,463 (3.0)	22,343 (99.5)	120 (0.5)	1.34 (1.11–1.60)	0.002	1.35 (1.12–1.62)	**0.002**
**BPH**							
No	730,672 (99.0)	727,733 (99.6)	2,939 (0.4)	1	–	1	–
Yes	7,196 (1.0)	7,148 (99.3)	48 (0.7)	1.66 (1.25–2.21)	<0.001	0.99 (0.74–1.33)	0.954
**CKD**							
No	669,698 (90.8)	667,039 (99.6)	2,659 (0.4)	1	–	1	–
Yes	68,170 (9.2)	67,842 (99.5)	328 (0.5)	1.21 (1.08–1.36)	0.001	0.71 (0.62–0.80)	**<0.001**
**COPD**							
No	734,756 (99.6)	731,788 (99.6)	2,968 (0.4)	1	–	1	–
Yes	3,112 (0.4)	3,093 (99.4)	19 (0.6)	1.52 (0.96–2.38)	0.07	0.92 (0.58–1.45)	0.720
**Dementia**							
No	734,399 (99.5)	731,426 (99.6)	2,973 (0.4)	1	–	–	–
Yes	3,469 (0.5)	3,455 (99.6)	14 (0.4)	1.00 (0.59–1.69)	0.991	–	–
**Depression**							
No	733,328 (99.4)	730,357 (99.6)	2,971 (0.4)	1	–	1	–
Yes	4,540 (0.6)	4,524 (99.6)	16 (0.4)	0.87 (0.53–1.42)	0.577	0.92 (0.56–1.51)	0.733
**Diabetes**							
No	636,738 (86.3)	634,268 (99.6)	2,470 (0.4)	1	–	1	–
Yes	101,130 (13.7)	100,613 (99.5)	517 (0.5)	1.32 (1.20–1.45)	<0.001	0.88 (0.79–0.98)	**0.024**
**Hyperlipidemia**							
No	568,241 (77.0)	566,284 (99.7)	1,957 (0.3)	1	–	1	–
Yes	169,627 (23.0)	168,597 (99.4)	1,030 (0.6)	1.77 (1.64–1.91)	<0.001	1.05 (0.95–1.17)	0.348
**Hypertension**							
No	575,677 (78.0)	573,704 (99.7)	1,973 (0.3)	1	–	1	–
Yes	162,191 (22.0)	161,177 (99.4)	1,014 (0.6)	1.83 (1.70–1.97)	<0.001	1.24 (1.11–1.38)	**<0.001**
**Hypothyroidism**							
No	725,107 (98.3)	722,196 (99.6)	2,911 (0.4)	1	–	1	–
Yes	12,761 (1.7)	12,685 (99.4)	76 (0.6)	1.49 (1.18–1.87)	0.001	1.19 (0.94–1.49)	0.147
**IHD**							
No	704,569 (95.5)	701,778 (99.6)	2,791 (0.4)	1	–	1	–
Yes	33,299 (4.5)	33,103 (99.4)	196 (0.6)	1.49 (1.29–1.72)	<0.001	0.97 (0.83–1.13)	0.654
**Osteoarthritis**							
No	674,781 (91.5)	672,227 (99.6)	2,554 (0.4)	1	–	1	–
Yes	63,087 (8.5)	62,654 (99.3)	433 (0.7)	1.82 (1.64–2.02)	<0.001	1.25 (1.12–1.38)	**<0.001**
**Osteoporosis**							
No	730,137 (99.0)	727,200 (99.6)	2,937 (0.4)	1	–	1	–
Yes	7,731 (1.0)	7,681 (99.4)	50 (0.6)	1.61 (1.22–2.13)	0.001	1.05 (0.79–1.40)	0.731
**Pre-diabetes**							
No	710,828 (96.3)	707,992 (99.6)	2,836 (0.4)	1	–	1	–
Yes	27,040 (3.7)	26,889 (99.4)	151 (0.6)	1.40 (1.19–1.65)	<0.001	0.92 (0.78–1.09)	0.338
**Stroke**							
No	721,752 (97.8)	718,836 (99.6)	2,916 (0.4)	1	–	–	–
Yes	16,116 (2.2)	16,045 (99.6)	71 (0.4)	1.09 (0.86–1.38)	0.470	–	–

Univariate analysis was performed to assess the association between each potential risk factor and HZ occurrence. Factors with *p* < 0.05 were included in the multivariate logistic regression model. Potential risk factors that showed an association with HZ occurrence in literature were also included regardless of the *p*-value. Hosmer-Lemeshow goodness-of-fit test, *Χ*^2^ = 5.68, df = 8, *p* = 0.684. OR, Odds Ratio; CI, Confidence interval; BPH, Benign Prostatic Hyperplasia; CKD, Chronic Kidney Disease; COPD, Chronic Obstructive Pulmonary Disease; IHD, Ischemic Heart Disease; IQR, Interquartile Range. The bolded values are those that are statistically significant (*p*<0.05).

## Discussion

In this retrospective database review, the HZ prevalence was 221, 224, and 203 per 100,000 persons in 2018, 2019, and 2020, respectively, with an average yearly prevalence of 216 per 100,000 persons. The mean and median ages of HZ diagnosis were 59.9 and 62.0 years. HZ prevalence peaked in the 70 to 79-year age group. Most individuals consulted only once (84.3%) and 8.9% required referrals. Oral acyclovir and topical acyclovir were prescribed in 71.6% and 47.6%, respectively. The commonest analgesia was gabapentin. Chinese and Malay ethnicities, asthma, hypertension, and osteoarthritis were associated with increased odds of HZ.

### Disease burden

The reduction in HZ prevalence in 2020 may be related to a decrease in respiratory infections resulting from Covid-19 control measures and a shift in health-seeking behavior. A Singaporean study reported that mandatory mask-wearing, social distancing, and lockdowns led to a reduced prevalence of multiple respiratory viruses in 2020 (41). Physical stress is a risk factor for HZ and coinfections may contribute to varicella zoster virus (VZV) reactivation (24). Hence, the dip in HZ prevalence could have mirrored that of respiratory infections by reducing reactivation triggers. An English study presented a 27% decrease in HZ cases during the peak pandemic period in 2020 compared to the same period in 2019 (42). The authors postulated that the incidence of non-Covid-19 conditions reduced with pandemic control measures, and health-seeking behavior changed due to fears of contracting Covid-19in healthcare settings.

Similar age-related patterns of HZ prevalence were demonstrated in the literature. A study on the global epidemiology of HZ reported the mean age of diagnosis as 59.4 years (1). A systematic review of Asia-Pacific countries established that HZ incidence peaked between 70 and 79 years, decreasing in those aged 80 years and above (5). While age-specific risks are a key factor in decisions regarding HZ vaccination, other factors such as age-specific vaccine efficacy and complication rates require careful consideration (43).

### Healthcare utilization

The number of visits for HZ was low, with most individuals consulting only once. In comparison, the mean number of outpatient visits per HZ episode ranged from 1 to 5.7 in Japan, South Korea, and Thailand (13–15, 18). In Japan, the higher number of visits was explained by differences in the healthcare system and the use of outpatient neurotropin injections for the treatment of post-herpetic neuralgia (15). The low number of visits observed may be explained by early presentation and treatment with oral antivirals. Prompt treatment of HZ with oral acyclovir reduces the time to pain resolution by more than 50% (44), and the time to 50% lesion healing by about 2.5 days (45). With appropriate early treatment, complication rates and the need for further healthcare visits may be reduced.

Similarly, reduction of complications via early effective antiviral therapy could explain the low referral rate in this study. The main reasons for referrals were to evaluate and manage complications, control zoster pain, or closely monitor high-risk patients. However, individuals with HZ may have consulted other healthcare providers for referrals, which would not be captured in the results.

Topical acyclovir was prescribed for almost half of individuals with HZ, but there is a lack of evidence to support this practice. Guidelines on the management of HZ have stated that topical antiviral therapy lacks efficacy in patients with HZ and is not recommended (46). A randomized controlled trial also demonstrated that topical acyclovir did not improve rash progression, pain severity, or the incidence of post-herpetic neuralgia (47). One possible reason for this practice is that patients sought topical acyclovir treatment after learning about it from sources other than their healthcare providers. Physician-prescribing decisions are substantially affected by patient requests (48). Physicians’ lack of knowledge of the limited evidence for topical acyclovir use in HZ may be another contributing factor. Future studies should explore the reasons for, benefits, and harms of this prescribing pattern.

The analgesia prescribed in this study was appropriate, but the use of analgesia combinations can be explored further. First-line treatment options for HZ-related pain are gabapentinoids and TCAs, while opioids and tramadol are second- or third-line options (49). Gabapentin reduced pain severity by 66% compared to 33% with placebo (50). Recent evidence has shown that combining gabapentin with a lidocaine patch has better analgesic effect than gabapentin alone, and a lower incidence of adverse events (51). However, only 2.9% of individuals with HZ were prescribed topical lidocaine. Primary care physicians may consider the use of combination therapy for patients who do not respond adequately to gabapentin alone.

### Associated factors

The detection of associations may generate hypotheses for further studies to identify high-risk individuals for HZ vaccination. A preponderance of HZ cases was identified among individuals of Chinese and Malay ethnicities. Little is known about ethnic differences in HZ occurrence, which may be explained by differences in genetic risk and healthseeking behaviors. A Malaysian study showed that Malays were more likely to consult a doctor for health issues than Indians (52).

The relationship demonstrated between asthma and HZ is consistent with other studies. In a meta-analysis, the pooled relative risk (RR) of HZ in a patient with asthma was 1.25 (95% CI 1.13–1.39) (24). Asthma, a Th2-predominant condition, results in reciprocal suppression of Th1 cell-mediated immunity (25), which may lead to VZV reactivation (53). The risk of HZ is even higher if asthmatic individuals are using inhaled or oral corticosteroids (26, 28). Systemic steroid use increases the risk of HZ by 59% (54), reflecting the importance of good asthma control.

Similar findings between hypertension and HZ have been reported. A Japanese study established a hazard ratio of 1.93 (95% CI 1.66–2.26) for HZ among hypertensive patients (35). A database review also demonstrated that hypertension was independently associated with HZ among patients with rheumatoid arthritis (55). T cell activation underlies the pathogenesis of hypertension (56). Although the mechanism underlying the observed association is unclear, it is suspected to be a decline in cell-mediated immunity (35).

Individuals with osteoarthritis had higher odds of HZ. A case–control study revealed a similar association between osteoarthritis and HZ (OR 1.24, 95% CI 1.20–1.28). A prospective study reported a higher incidence of HZ among individuals with non-inflammatory musculoskeletal disorders (57). Cell-mediated immunity is generally impaired in patients with chronic illnesses (58). A study comparing healthy and “almost healthy” persons defined by the lack of exercise or by the use of medications for chronic conditions such as hypertension or osteoarthritis demonstrated lower levels of interleukin 2 and higher levels of interleukin 6 in the latter (59).

CKD and diabetes were negatively associated with HZ. A prospective cohort study demonstrated that patients with diabetes were less likely to develop HZ (HR 0.7, 95% CI 0.5–1.0) (57), for reasons unknown. A systematic review established that evidence of diabetes as a risk factor for HZ was inconclusive (5). A possible reason for the negative association with HZ is that individuals with CKD and diabetes may have regular follow-ups with other healthcare providers and increased opportunities for HZ vaccination, thereby reducing the risk of contracting HZ. Another postulation for the observed relationship between CKD and HZ is effect modification by CKD subgroups. CKD is diagnosed via markers of kidney damage (urinary indices, imaging) or reduced glomerular filtration rate for more than 3 months, as per the Kidney Disease: Improving Global Outcomes 2012 Clinical Practice Guideline (60). Literature is scarce regarding the risk of HZ in different CKD stages or diagnosis groups. However, subgroup analysis was not performed due to limited data availability regarding how CKD diagnoses were established.

### Strengths and limitations

This study is one of few population-based studies that have examined the burden of HZ and healthcare utilization in a primary care setting. There has been a limited number of epidemiological studies on HZ implemented in primary care, which is significant as vaccinations are mostly administered in primary care settings.

Since patient visits to public primary care clinics in other clusters, as well as private clinics, could not be captured in the eHints database, the prevalence of HZ, healthcare utilization rates, and associated factors such as chronic conditions may be underestimated. Individuals who chose to self-medicate instead of seeking medical attention would contribute to underreporting of cases.

In examining the association between factors of interest and HZ, the varying clinical profiles of patients who sought care from different healthcare providers may have confounded results. The differences in patient profiles and practices of public and private care limit the generalizability of findings to the private arm of primary care.

The duration from the onset of HZ symptoms to presentation at the first outpatient visit was unknown. This duration would have influenced whether oral antiviral therapy was prescribed (i.e., the proportion of cases prescribed oral antiviral therapy), the effectiveness of oral antiviral therapy, and the subsequent likelihood of complications and referral rate.

Due to limited data availability, all HZ visits for each case were assumed to belong to a single HZ episode. Therefore, recurrent HZ episodes would be missed. Nevertheless, recurrence often occurs many years after the initial episode (61). Less than 1% of cases in this study had HZ visits spaced three or more months apart, suggesting that the assumption above was less likely to affect the results.

## Conclusion

The prevalence of HZ in this study ranged from 203 to 224 per 100,000 persons, with the highest prevalence in the 70 to 79-year age group. Oral acyclovir and topical acyclovir were prescribed in 71.6% and 47.6%, respectively. The most common analgesia used was gabapentin. The median number of primary care visits per HZ episode was 1% and 8.9% of individuals required referrals for management beyond primary care. A median of 5 days of medical leave was required per HZ episode among working individuals.

The findings of this study suggest a need for HZ prevention among the older age groups. Visitation and referral rates were low. The use of topical acyclovir was uncovered, and further research should explore the rationale, benefits, and harms of topical acyclovir, as well as the use of analgesia combinations in individuals with HZ who do not respond adequately to single analgesia.

## Data availability statement

The datasets presented in this article are not readily available because their use is subject to restrictions under the SingHealth Data Access Policy. Requests to access the datasets should be directed to SingHealth Polyclinics Data Protection Office (Email: shppdpa@singhealth.com.sg).

## Ethics statement

Ethical approval was not required for the study involving humans in accordance with the local legislation and institutional requirements. Written informed consent to participate in this study was not required from the participants or the participants’ legal guardians/next of kin in accordance with the national legislation and the institutional requirements.

## Author contributions

X-BC, NT, CJN, and CWN conceived and designed the study. WA provided input on the type of data available for extraction during the design phase. X-BC, DN, and YK conducted data cleaning, merging, and analysis. X-BC drafted the manuscript. All authors contributed to the article and approved the submitted version.

## Conflict of interest

The authors declare that the research was conducted in the absence of any commercial or financial relationships that could be construed as a potential conflict of interest.

## Publisher’s note

All claims expressed in this article are solely those of the authors and do not necessarily represent those of their affiliated organizations, or those of the publisher, the editors and the reviewers. Any product that may be evaluated in this article, or claim that may be made by its manufacturer, is not guaranteed or endorsed by the publisher.
