# Supplementary material for: Prevalence and healthcare utilization in managing herpes zoster in primary care: a retrospective study in an Asian urban population
Source: Front Public Health. 2023 Sep 15;11:1213736. doi: 10.3389/fpubh.2023.1213736 (PMC10540814; doi:10.3389/fpubh.2023.1213736)
Supplement: Supplementary file 1 [file Table_1.DOCX]

Supplementary Material

Prevalence and Healthcare Utilization in Managing Herpes Zoster in Primary Care: A Retrospective Study in an Asian Urban Population

Xin-Bei Valerie Chan^1,2*^, Ngiap Chuan Tan^1,2^, Chung Wai Mark Ng^1,2^, Ding Xuan Ng^1^, Yi Ling Eileen Koh^1^, Wai Keong Aau^1^, Chirk Jenn Ng^1,2^

^1^SingHealth Polyclinics, Singapore

^2^SingHealth Duke-NUS Family Medicine Academic Clinical Programme, Singapore

*** Correspondence:**Xin-Bei Valerie Chan
[valerie.chan.x.b@singhealth.com.sg](mailto:valerie.chan.x.b@singhealth.com.sg)

**Table A-1.** List of ICD10 Codes used to define Herpes Zoster and studied Chronic Conditions

| **Condition** | **ICD 10 Code** | **ICD 10 Descriptor** |
| --- | --- | --- |
| Anxiety | F41.9 | Anxiety disorder, unspecified |
| Asthma | J45.9 | Asthma, unspecified |
| Benign Prostatic Hyperplasia | N40 | Benign prostatic hyperplasia |
| Bipolar Disorder | F31.9 | Bipolar disorder, unspecified |
| Chronic Kidney Disease | N18.2 | Chronic kidney disease, stage 2 (mild) |
|  | N18.3 | Chronic kidney disease, stage 3 (moderate) |
|  | N18.4 | Chronic kidney disease, stage 4 (severe) |
|  | N18.5 | Chronic kidney disease, stage 5 |
|  | N18.9 | Chronic kidney disease, unspecified |
|  | N05.9 | Unspecified nephritic syndrome with unspecified morphologic changes |
|  | N06.9 | Isolated proteinuria with unspecified morphologic lesion |
|  | E11.21 | Type 2 diabetes mellitus with diabetic nephropathy |
|  | E11.22 | Type 2 diabetes mellitus with diabetic chronic kidney disease |
| Chronic Obstructive Pulmonary Disease | J44.9 | Chronic obstructive pulmonary disease, unspecified |
| Dementia | F03 | Unspecified dementia |
| Depression | F32.9 | Depressive episode, unspecified |

**Table A-1.** List of ICD10 Codes used to define Herpes Zoster and studied Chronic Conditions (continued)

| **Condition** | **ICD 10 Code** | **ICD 10 Descriptor** |
| --- | --- | --- |
| Diabetes Mellitus | E10.9 | Type 1 diabetes mellitus without complications |
|  | E11.9 | Type 2 diabetes mellitus without complications |
|  | E11.21 | Type 2 diabetes mellitus with diabetic nephropathy |
|  | E11.22 | Type 2 diabetes mellitus with diabetic chronic kidney disease |
|  | E11.3 | Type 2 diabetes mellitus with ophthalmic complications |
|  | E11.40 | Type 2 diabetes mellitus with diabetic neuropathy, unspecified |
|  | E11.621 | Type 2 diabetes mellitus with foot ulcer |
| Epilepsy | G40.9 | Epilepsy, unspecified |
| Herpes Zoster | B02.9 | Zoster, not otherwise specified (NOS) |
| Hyperlipidemia | E78.0 | Pure hypercholesterolemia |
|  | E78.5 | Hyperlipidemia, unspecified |
| Hypertension | I10 | Essential (primary) hypertension |
| Hypothyroidism | E03.9 | Hypothyroidism, unspecified |
| Ischemic Heart Disease | I25.9 | Chronic ischemic heart disease, unspecified |
| Osteoarthritis | M15.9 | Polyosteoarthritis, unspecified |
| Osteoporosis | M80.9 | Unspecified osteoporosis with pathological fracture |
|  | M81.9 | Osteoporosis, unspecified |
| Parkinson’s Disease | G20 | Parkinson's disease |
| Prediabetes | R73.01 | Impaired fasting glucose |
|  | R73.02 | Impaired glucose tolerance (oral) |
| Psoriasis | L40.0 | Psoriasis vulgaris |
| Rheumatic Arthritis | M06.9 | Rheumatoid arthritis, unspecified |
| Schizophrenia | F20.9 | Schizophrenia, unspecified |
| Stroke | I64 | Stroke, not specified as hemorrhage or infarction |
